# Same‐day Hyaluronic Acid rectal spacer for TRUS‐guided HDR Prostate Brachytherapy: Feasibility and dosimetric outcomes

**DOI:** 10.1002/acm2.70701

**Published:** 2026-07-08

**Authors:** Sook Kien Ng, Damodar Pokhrel, Colin Huang, Francis Adumata Asamoah, Jordan Alexander Holmes, Arpan Prabhu, Omar Ishaq

**Affiliations:** ^1^ Department of Radiation Oncology Indiana University School of Medicine Indianapolis Indiana USA

**Keywords:** HDR prostate brachytherapy, rectal dose, rectal spacer, TRUS‐guided

## Abstract

**Background:**

Rectal spacers are widely used to reduce rectal dose in prostate radiotherapy. However, their use during transrectal ultrasound (TRUS)‐guided high‐dose‐rate (HDR) brachytherapy has been limited by degradation of ultrasound image quality when placed on the day of the procedure, often necessitating CT‐based planning or separate‐day workflows.

**Purpose:**

To evaluate the feasibility and dosimetric impact of same‐day hyaluronic acid (HA) rectal spacer injection in patients undergoing TRUS‐guided HDR prostate brachytherapy.

**Methods:**

Following institutional review board approval, a retrospective analysis was performed on 20 patients with clinically localized prostate cancer treated with HDR brachytherapy combined with external beam radiation therapy between April 2023 and March 2024. Ten patients with a prostate–anterior rectal wall distance < 5 mm received HA rectal spacer injection immediately prior to HDR brachytherapy, while ten patients with a distance > 5 mm did not undergo spacer placement. HA spacer (3–6 cc) was injected into the perirectal space under sagittal TRUS guidance, followed by catheter placement and real‐time TRUS‐based treatment planning. A single fraction of 15 Gy was delivered. Dosimetric parameters were compared using an unpaired two‐tailed Student's *t*‐test.

**Results:**

HA rectal spacer placement was technically successful in all patients (10/10) and preserved ultrasound image quality for catheter placement and treatment planning. Spacer placement improved prostate V100 (95.6% ± 0.5% vs. 94.5% ± 0.6%; *p* < 0.001) and significantly reduced rectal V75 (0.42 ± 0.25 cc vs. 0.97 ± 0.34 cc; *p* < 0.001). No significant differences were observed in urethral D10 or bladder D0.1cc.

**Conclusions:**

Same‐day HA rectal spacer injection is feasible during TRUS‐guided HDR prostate brachytherapy and preserves ultrasound image quality, enabling fully real‐time treatment planning without the need for CT‐based workflows. This approach provides rectal dose reduction while maintaining target coverage and may offer a streamlined alternative to multistep spacer placement strategies.

## 1 INTRODUCTION

Prostate brachytherapy (PBT) stands as a definitive treatment for prostate cancer.^1^, ^2^, ^3^ High‐dose‐rate (HDR) PBT can either be used alone or in conjunction with external beam radiotherapy (EBRT) to treat low, intermediate, and high‐risk prostate cancer cases.^4^, ^5^, ^6^ It is imperative to minimize radiation doses to the bladder, urethra, and rectum to preserve patients’ quality of life.^7^, ^8^, ^9^, ^10^ Elevated radiation exposure to the rectum heightens the risk of gastrointestinal toxicity.^7^, ^8^, ^9^, ^10^ An effective strategy to limit rectal toxicity is to expand the distance between the rectum and prostate using a rectal spacer.^11^, ^12^, ^13^, ^14^, ^15^ Even marginal increases in this distance lead to significant reductions in rectal dose due to the rapid dose fall‐off with PBT.^12^, ^13^, ^14^, ^15^, ^16^, ^17^

Numerous studies have evaluated the use of polyethylene glycol (PEG) rectal spacers placed between the anterior rectal wall and prostate to improve dosimetry and reduce rectal toxicity during HDR PBT.^13^, ^15^, ^16^, ^18^ However, PEG spacers can impair prostate visualization on real‐time ultrasound images immediately after injection, potentially complicating catheter implantation and TRUS‐based treatment planning. For this reason, CT‐based planning is often used when PEG spacers are inserted on the day of the brachytherapy procedure.^13^, ^15^, ^16^ One advantage of TRUS‐based planning compared with CT‐based planning is that it eliminates the need for patient transfer between catheter placement, treatment planning, and treatment delivery, thereby reducing the risk of catheter displacement and improving workflow efficiency.^2^ To maintain ultrasound visualization when PEG spacers are used, spacer placement is typically performed several days prior to brachytherapy to allow gas pockets to dissipate and image quality to recover.^18^, ^19^ However, this approach requires an additional procedure on a separate day, which may increase logistical complexity, procedural risk, and cost.

Recently, hyaluronic acid (HA) rectal spacer (Barrigel™, Palette Life Sciences, Carlsbad, CA, USA) has been used to reduce rectal dose in prostate EBRT ^20^, ^21^, ^22^ and BT ^23^, ^24^ by increasing separation between the prostate and rectum. Compared with PEG‐based spacers, HA spacers may preserve ultrasound image quality during placement and treatment planning, potentially facilitating same‐day spacer insertion during TRUS‐guided HDR brachytherapy workflows.

The objective of this study was to compile data from patients who underwent HA rectal spacer injection immediately before HDR PBT and compare it with an earlier group of patients without rectal spacer injection. Our hypothesis was that the increase in separation between the prostate gland and anterior rectal wall would achieve reduced rectal dose and improve overall dosimetry of HDR PBT plans.

## 2 MATERIALS AND METHODS

### 2.1 Patient characteristics

After institutional review board approval, treatment records were reviewed for 20 men with clinically localized prostate cancer treated with radiotherapy at a single institution between April 2023 and March 2024—including 10 patients who received a rectal spacer injection immediately before HDR brachytherapy and 10 without. Patients were included if they received definitive HDR brachytherapy plus EBRT and had a prostate–anterior rectal wall distance of less than 5 mm. Patients were excluded from rectal HA spacer placement if the prostate–anterior rectal wall distance exceeded 5 mm or if prostate volume was greater than 55 cc, as measured on US images obtained at the start of the brachytherapy procedure before catheter insertion.

### 2.2 HA rectal spacer injection

During the HDR brachytherapy procedure, patients were positioned in the dorsal lithotomy position with their legs in stirrups under general anesthesia. To increase the natural anatomic space between the prostate and rectum, a standard‐to‐low lithotomy leg position (without excessive leg elevation) was used. A foley catheter was inserted, rectal preparation was confirmed, and the perineum was prepped with betadine. A TRUS probe, affixed to a brachytherapy US stepper, was inserted into the rectum to visualize critical anatomy. The probe was adjusted to obtain optimal contact with the prostate, which enhanced the quality of imaging.

The initial set of US images was captured, and the distance between the prostate and the anterior rectal wall was measured. For patients with a prostate and rectal wall distance of less than 5 mm, HA rectal spacer was injected into the perirectal space. Prior to injection, the 18G needle used for spacer injection was primed with HA gel to remove all air. The needle was then inserted into the perineum, using a freehand technique under sagittal TRUS imaging. The first of three syringes, each containing 3 cc of HA gel, was injected using a freehand technique into the perineum, posterior to Denonvilliers’ fascia and anterior to the rectal wall, to displace the rectal wall posteriorly. Care was taken during injection to ensure that prostate elevation did not cause pubic arch obstruction, which could complicate HDR catheter placement. If additional separation was required, an additional 3 cc of HA gel was injected. Laterality of prostate displacement was verified via axial TRUS imaging.

### 2.3 HDR implant and treatment planning

Following injection of the HA rectal spacer, HDR catheters were inserted transperineally into the prostate using a template technique under TRUS guidance. The ProGuide™ (Nucletron, Veenendaal, Netherlands) HDR brachytherapy catheters were implanted from anterior to posterior in a modified peripheral loading pattern. Catheter positions and insertion depths were verified on transverse and sagittal TRUS views to confirm adequate geometry for achieving dosimetric objectives, after which the template was locked. A final axial TRUS dataset was then acquired at 1 mm slice spacing and used to reconstruct a three‐dimensional US volume for treatment planning. A single radiation oncologist contoured the clinical target volume (prostate gland and involved seminal vesicles, when applicable) and organs at risk, including the anterior rectal wall, urethra, and bladder, in accordance with GEC‐ESTRO recommendations.^25^, ^26^, ^27^ The TRUS‐based inverse treatment planning was performed using the Oncentra Prostate (v4.2, Elekta AB, Stockholm, Sweden) treatment planning system.

The medical physicists then generated virtual catheter paths on the reconstructed US volume and confirmed that the virtual catheter trajectories and insertion depths matched those of the implanted catheters. Source dwell positions located within the clinical target volume were activated, and dwell times were calculated using inverse optimization within Oncentra Prostate software. A treatment plan was generated to deliver a single‐fraction prescription dose of 15 Gy while meeting the following dosimetric objectives: prostate V100 > 95%, prostate V150 < 35%, prostate V200 < 12%, urethra D10 < 118%, and rectum V75 < 0.5 cc. Dose–volume histograms (DVHs) were reviewed for the clinical target volume and adjacent organs at risk including rectal wall. The radiation oncologists performed the final plan evaluation and when necessary adjusted anatomy‐oriented dose optimization using the Graphical Optimization Tool in Oncentra Prostate Software, interactively refining the isodose distributions on a slice‐by‐slice basis.

After all dosimetric objectives were satisfied and the HDR plan was approved by the treating physician, an independent secondary check was performed by the second experienced physicist using commercially available RadCalc (v7.3, Lifeline Software, Inc., a LAP Group company). This check verified key treatment parameters, including source strength, reference date, treatment date, number of catheters, number of dwell positions, and the distance to the first dwell position. In addition, two dose points, manually selected in low‐dose gradient regions, were confirmed to agree within ± 3% of the original calculation. Final DVHs were then generated, and doses to the prostate and organs at risk were recorded.

### 2.4 Treatment delivery

**FIGURE 1:**
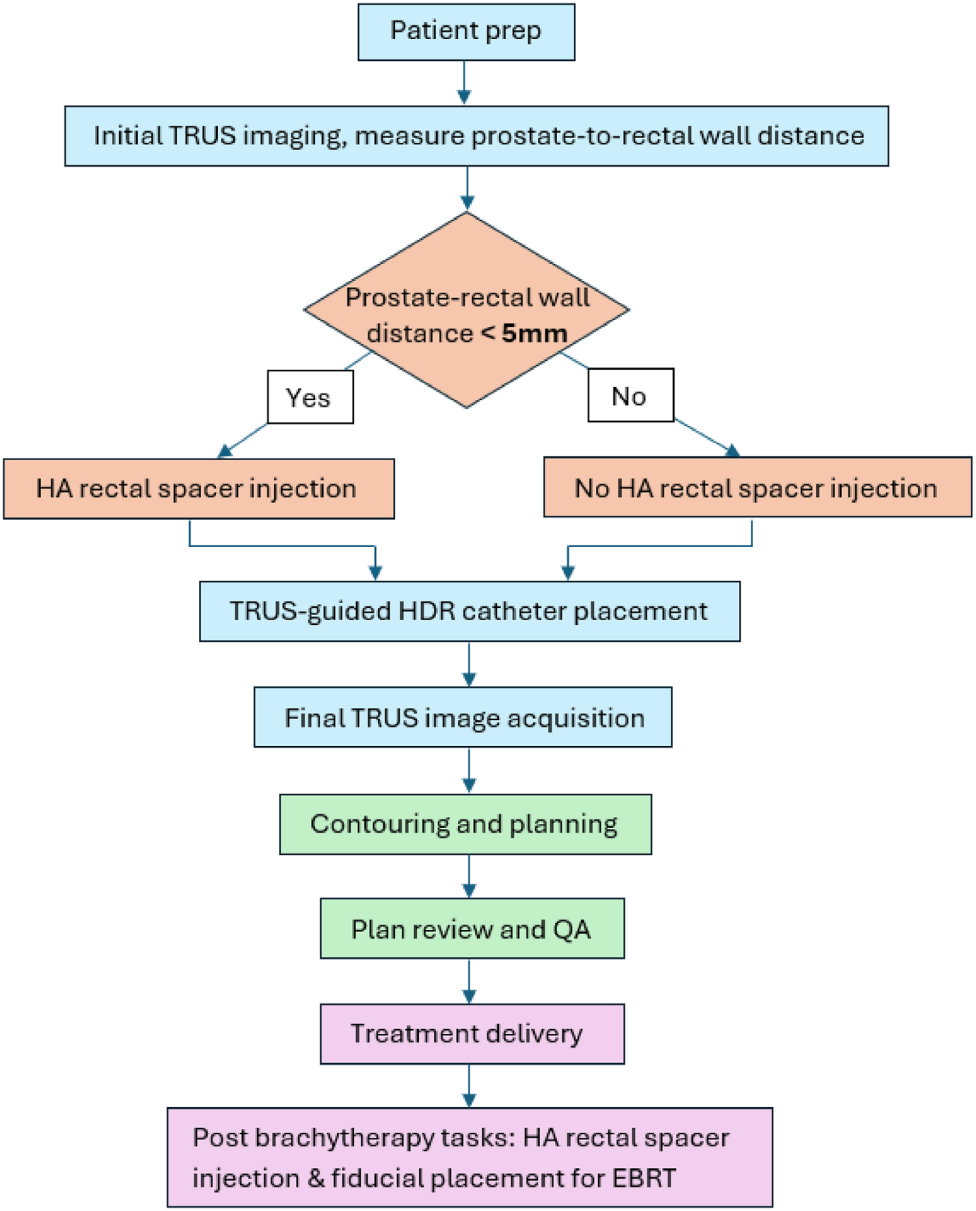
Schematic workflow of same‐day HA rectal spacer placement for TRUS‐guided HDR prostate brachytherapy. Patients with a prostate‐to‐anterior rectal wall distance of less than 5 mm received 3–6 cc of HA spacer before HDR catheter placement, while those with a distance greater than 5 mm did not receive pre‐HDR spacer injection. HDR catheters were then placed under TRUS guidance, followed by image acquisition, contouring, treatment planning, plan review, QA, and treatment delivery with the Flexitron remote afterloader. After HDR brachytherapy delivery, the remaining HA rectal spacer was injected in patients who received an initial rectal spacer injection, whereas patients without pre‐HDR rectal spacer placement receive the full 9 cc post‐HDR brachytherapy.

The finalized HDR prostate treatment plan was electronically transferred from the treatment planning system to the Flexitron remote afterloader treatment console (Elekta AB, Stockholm, Sweden). Safety checks and quality assurance procedures were performed by the medical physicists and radiation oncologist to verify accurate plan transfer and afterloader functionality. Treatment delivery was then executed using the Flexitron remote afterloader. Following HDR brachytherapy delivery, additional HA rectal spacer gel was injected as needed to augment perirectal spacing and minimize rectal dose during subsequent EBRT. In addition, in the same set up, gold fiducial markers were also transperineally inserted into the left and right mid‐lateral prostate and prostatic apex to enable prostate localization via electronic portal imaging prior to each EBRT fraction. The schematic workflow for same‐day HA rectal spacer placement in TRUS‐guided HDR PBT is shown in Figure 1.

### 2.5 Workflow for patients excluded from HA rectal spacer placement

For patients excluded from HA rectal spacer placement prior to HDR PBT, the workflow was as follows. Patients with a prostate–anterior rectal wall distance greater than 5 mm were not considered for HA rectal spacer injection before HDR brachytherapy. Aside from omission of the spacer placement step, the subsequent HDR PBT procedural workflow was identical to that used for patients who received the HA rectal spacer as described before.

### 2.6 Data collection and statistical analysis

Dosimetric parameters for the prostate and organs at risk, including the rectum, bladder, and urethra, were collected for both patient groups. Mean values were compared between patients with and without HA spacer placement using an unpaired two‐tailed Student's *t*‐test. The dosimetric variables demonstrated approximately symmetric distributions without substantial outliers, and variances between groups were comparable; therefore, parametric analysis was considered appropriate. To assess the robustness of the findings independent of distributional assumptions, additional non‐parametric comparisons were performed using the Mann–Whitney *U* test. The non‐parametric analysis yielded conclusions identical to those obtained with the Student's *t*‐test. A *p*‐value less than 0.05 was considered statistically significant.

## 3 RESULTS

Radiation dose parameters for the prostate and organs at risk for patients receiving HA rectal spacers are summarized in Table 1, while corresponding results for patients without spacer placement are shown in Table 2. HA rectal spacer placement was technically successful in all cases (10/10). Two patients received 6 cc of HA spacer prior to HDR brachytherapy, while the remaining patients received 3 cc before catheter placement. After completion of HDR treatment, additional HA gel was injected as needed so that all patients ultimately received a total of 9 cc of HA spacer to maintain rectal separation during subsequent EBRT.

**TABLE 1:** The radiation doses to the prostate gland and organs at risk for the patient group receiving the HA rectal spacer prior to HDR PBT.

Pt no.	Prostate volume (cc)	Prostate V90 (%)	Prostate V100 (%)	Prostate V150 (%)	Prostate V200 (%)	Bladder D0.1cc (Gy)	Rectal V75 (cc)	Urethra D10 (%)
1	53.33	99.13	95.72	25.3	7.59	15.58	0.39	110.79
2	37.05	98.74	95.12	29.83	8.72	11.35	0.37	111.2
3	50.48	98.79	95.5	32.67	8.85	14.14	0.73	110.5
4	38.26	98.99	96.23	33.04	9.33	11.98	0.02	110.68
5	40.88	99.18	96.02	32.26	11.09	11.86	0.29	115.53
6	28.43	98.71	95.02	30.69	10.76	13.24	0.47	111.87
7	58.12	99.32	96.59	33.04	10.05	10.63	0.47	110.46
8	28.14	99.27	95.64	33.69	13.84	13.07	0.13	114.58
9	48.06	98.58	95.01	35.24	11.81	14.5	0.52	116.82
10	45.58	98.77	95.36	34.00	11.51	16.04	0.83	113.73
*Average*	*42.83*	*98.95*	*95.62*	*31.98*	*10.36*	*13.24*	*0.42*	*112.62*
*Std Dev*	*10.10*	*0.26*	*0.53*	*2.82*	*1.83*	*1.81*	*0.25*	*2.36*
*Median*	*43.23*	*98.89*	*95.57*	*32.86*	*10.41*	*13.16*	*0.43*	*111.54*

**TABLE 2:** The radiation doses to the prostate gland and organs at risk for the patient group without HA rectal spacer injection prior to HDR PBT.

Pt no.	Prostate volume (cc)	Prostate V90 (%)	Prostate V100 (%)	Prostate V150 (%)	Prostate V200 (%)	Bladder D0.1cc (Gy)	Rectal V75 (cc)	Urethra D10 (%)
1	32.79	98.84	95.18	32.66	12.45	12.92	0.80	115.86
2	23.68	98.69	94.01	34.96	12.56	14.55	0.75	111.32
3	38.81	98.69	94.23	31.48	9.90	13.38	0.60	110.93
4	44.06	99.06	93.68	31.95	12.55	14.24	1.18	112.55
5	26.38	98.80	95.02	30.10	9.78	13.27	0.84	110.97
6	35.32	98.91	94.20	36.41	13.42	11.87	0.63	110.89
7	67.56	99.09	94.10	27.43	9.56	14.66	0.77	112.72
8	46.33	99.22	95.27	29.46	10.76	13.42	1.19	111.26
9	50.75	98.82	95.00	33.13	10.94	13.01	1.35	113.71
10	33.79	98.73	94.14	35.28	14.13	13.94	1.61	116.39
*Average*	*39.95*	*98.89*	*94.48*	*32.29*	*11.61*	*13.53*	*0.97*	*112.66*
*Std Dev*	*12.90*	*0.18*	*0.57*	*2.81*	*1.62*	*0.85*	*0.34*	*2.06*
*Median*	*37.07*	*98.83*	*94.22*	*32.31*	*11.70*	*13.40*	*0.82*	*111.94*

**FIGURE 2:**
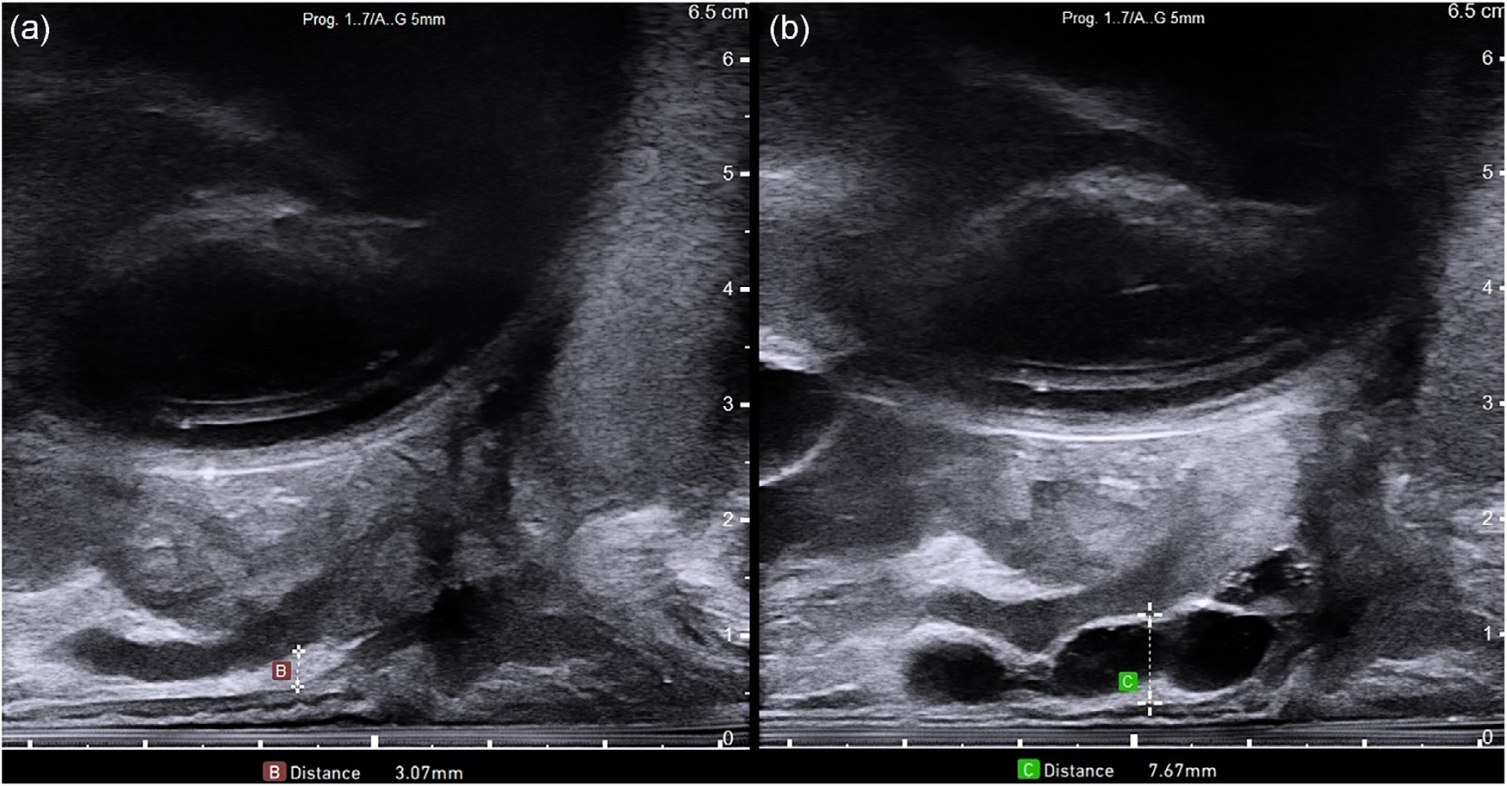
(a) A sagittal section of a TRUS image showing the distance between the anterior rectal wall and the prostate to be 3.07 mm before HA spacer injection. (b) A sagittal section of the same anatomy after 3 cc HA rectal spacer injection, demonstrating that the HA rectal spacer (hypoechoic area) has displaced the rectum posteriorly away from the prostate. The 3 cc HA rectal spacer injection increased the distance between the anterior rectal wall and the prostate by 4.6 mm.

Priming the needle used for HA spacer injection effectively removed all air, ensuring that the injection procedure did not introduce air into the injection site. Consequently, HA rectal spacer injection did not cause artifacts in TRUS images. The HA spacer appeared as a hypoechoic (darker) area compared to the surrounding tissue, which appeared hyperechoic (lighter) in the TRUS images (Figure 2). After injection, the visibility of the prostate and surrounding critical anatomy remained optimal and did not interfere with catheter placement. Therefore, HA spacer injection prior to HDR brachytherapy was feasible for TRUS‐guided delivery.

**FIGURE 3:**
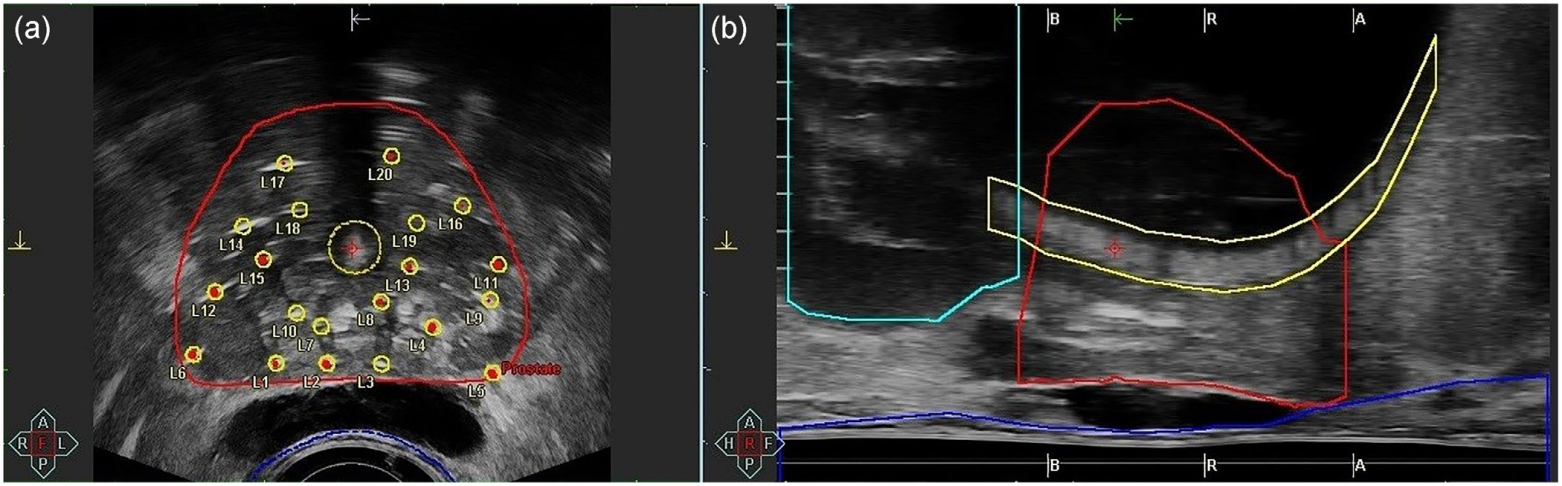
TRUS images demonstrate the HA rectal spacer placement for HDR prostate treatment planning. (a) Axial TRUS image showing the HA spacer creating adequate separation between the prostate gland (red contour) and anterior rectal wall (dark blue contour) while maintaining visualization of anatomical structures. (b) Sagittal TRUS image illustrating the craniocaudal extent of HA spacer placement between the prostate, rectum, bladder (light blue contour) and urethra (yellow contour).

Figure 3 demonstrates TRUS images acquired for treatment planning in a prostate cancer patient who received HA rectal spacer at our institution. Unlike the PEG spacer, the HA spacer maintains adequate acoustic transparency, allowing clear visualization of both the prostate gland and the created separation from the anterior rectal wall. The HA hydrogel exhibits acoustic properties more similar to soft tissue, minimizing impedance mismatch and reducing artifact generation. This preserved image quality enables accurate prostate contouring and target delineation on TRUS‐based treatment planning, while the spacer effectively increases the distance between the prostate and rectum, facilitating rectal dose sparing during radiation therapy.

As shown in Table 3, the use of HA rectal spacer resulted in a small but statistically significant increase in prostate V100 (95.6% ± 0.5% with spacer vs. 94.5% ± 0.6% without spacer; *p* < 0.001) in this cohort. No significant differences were observed in prostate V150 (32.0% ± 2.8% vs. 32.3% ± 2.8%; *p* = 0.81) or prostate V200 (10.4% ± 1.8% vs. 11.6% ± 1.6%; *p* = 0.12) between patients with and without spacer placement. However, the HA rectal spacer decreased rectal V75 significantly, (0.42 cc ± 0.25 cc with spacer vs. 0.97 cc ± 0.34 cc without spacer, *p* < 0.001). There was no significant difference in urethra and bladder radiation doses between the groups with and without HA rectal spacer, including urethra D10 (112.6% ± 2.4% with spacer vs. 112.7% ± 2.1% without spacer, *p* = 0.97) and bladder D0.1cc (13.22 Gy ± 1.8 Gy with spacer vs. 13.5 Gy ± 0.9 Gy without spacer, *p* = 0.66).

**TABLE 3:** Comparisons of the average dose indices between the patient group with and without HA rectal spacer injection prior to HDR PBT. Significant *p*‐values were highlighted in bold.

Average prostate volume and dose indices	With HA spacer	Without HA spacer	*p* value
Average prostate volume (cc)	42.83	39.95	0.584
Average Prostate V90 (%)	98.95	98.89	0.541
Average Prostate V100 (%)	95.62	94.48	**< 0.001**
Average Prostate V150 (%)	31.98	32.29	0.808
Average Prostate V200 (%)	10.36	11.61	0.123
Average rectal V75 (cc)	0.42	0.97	**< 0.001**
Average urethra D10 (%)	112.62	112.66	0.965

## 4 DISCUSSION

In this study, we describe our clinical workflow for performing real‐time TRUS‐guided HDR PBT with same‐day HA rectal spacer injection and report early dosimetric outcomes. To our knowledge, this is among the first reports evaluating the feasibility of HA rectal spacer placement immediately prior to TRUS‐guided HDR brachytherapy while preserving ultrasound image quality sufficient for fully real‐time TRUS‐based treatment planning. In this cohort, spacer placement was associated with reduced rectal dose and a modest improvement in prostate target coverage. However, it is important to recognize that the spacer cohort consisted of patients with initially limited prostate–rectal wall separation (< 5 mm), representing a more anatomically challenging group for achieving adequate target coverage while respecting rectal dose constraints. In patients with minimal prostate–rectal wall distance and without HA spacer placement, prostate dose coverage may need to be reduced in order to limit rectal dose. Therefore, the ability to modestly improve prostate coverage while simultaneously reducing rectal dose in this cohort supports the potential clinical utility of same‐day HA spacer placement.

Several previously published HDR PBT studies performed without rectal spacers have demonstrated that rectal dose remains an important dosimetric limitation, particularly in patients with minimal baseline prostate–rectal separation. Snyder et al.^7^ and Keyes et al.^8^ demonstrated correlations between rectal dose‐volume parameters and the risk of radiation‐related rectal toxicity following PBT. Similarly, studies evaluating HDR brachytherapy boost techniques have emphasized the importance of minimizing rectal dose while maintaining adequate prostate coverage.^4^, ^5^, ^6^ In standard HDR workflows without spacer placement, optimization of rectal dose constraints may require compromises in catheter geometry or target dose coverage, especially in patients with smaller anterior–posterior prostate dimensions or limited prostate–rectal spacing.

**FIGURE 4:**
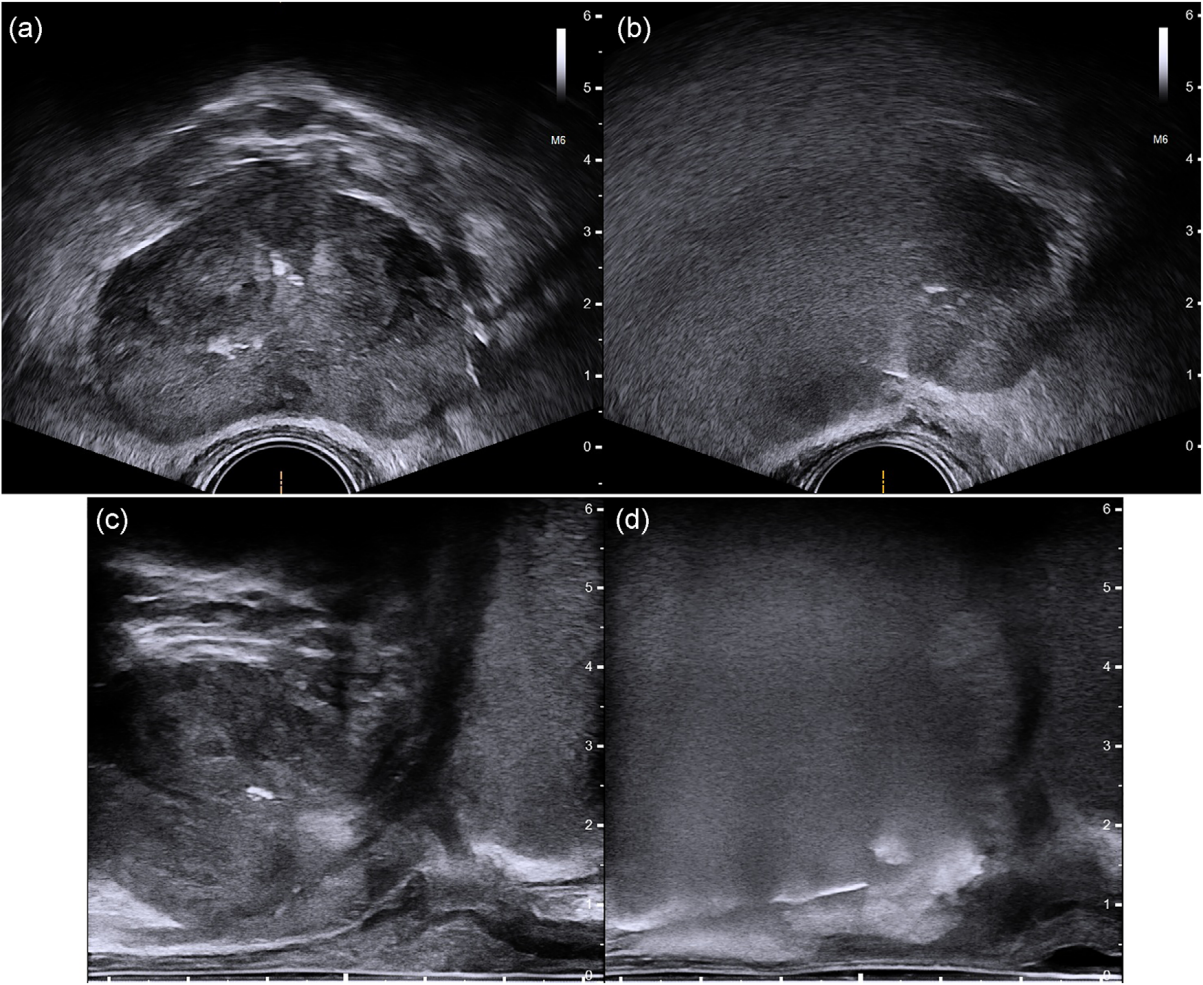
TRUS images demonstrating the impact of PEG rectal spacer on prostate visualization. (a) Axial TRUS image of the prostate mid‐gland before spacer injection showing clear anatomical detail. (b) Axial TRUS image immediately following PEG spacer injection demonstrating complete loss of prostate visualization. (c) The corresponding sagittal TRUS image before spacer injection. (d) Sagittal TRUS image after spacer injection showing acoustic artifacts and posterior shadowing obscuring the prostate gland boundary.

Our findings are consistent with prior reports evaluating rectal spacers during HDR PBT. Strom et al.^16^ demonstrated that hydrogel spacer placement significantly reduced rectal dose during HDR brachytherapy with or without IMRT, although CT‐based planning workflows were commonly required because immediate post‐injection ultrasound image quality was degraded. Similarly, Wu et al.^13^ and Chao et al.^15^ reported improved rectal dosimetry with hydrogel spacers during HDR brachytherapy, with reductions in rectal dose‐volume parameters while maintaining acceptable target coverage. However, in many previously reported workflows, spacer placement was performed on a separate day from brachytherapy or required CT‐based treatment planning because PEG spacer injection introduced ultrasound artifacts that interfered with prostate visualization. Figure 4(a–d) presents TRUS images of a prostate cancer patient who received PEG rectal spacer at our institution. TRUS images acquired before and immediately after PEG spacer injection demonstrate complete loss of prostate gland visualization following spacer placement. Air bubbles within the hydrogel mixture and residual air trapped during injection and mixing create extreme acoustic impedance mismatch, resulting in total acoustic reflection, strong specular echoes, and posterior acoustic shadowing. Additionally, material impedance mismatch at the spacer‐prostate and prostate‐rectal wall interfaces generates bright boundary artifacts, edge shadowing, and loss of anatomical detail due to differences in speed of sound propagation and acoustic attenuation. In contrast, our study demonstrates that same‐day HA spacer placement can be performed immediately before catheter implantation without compromising TRUS image quality, thereby preserving the advantages of real‐time TRUS‐guided planning and eliminating the need for additional procedures or patient transfers.

**FIGURE 5:**
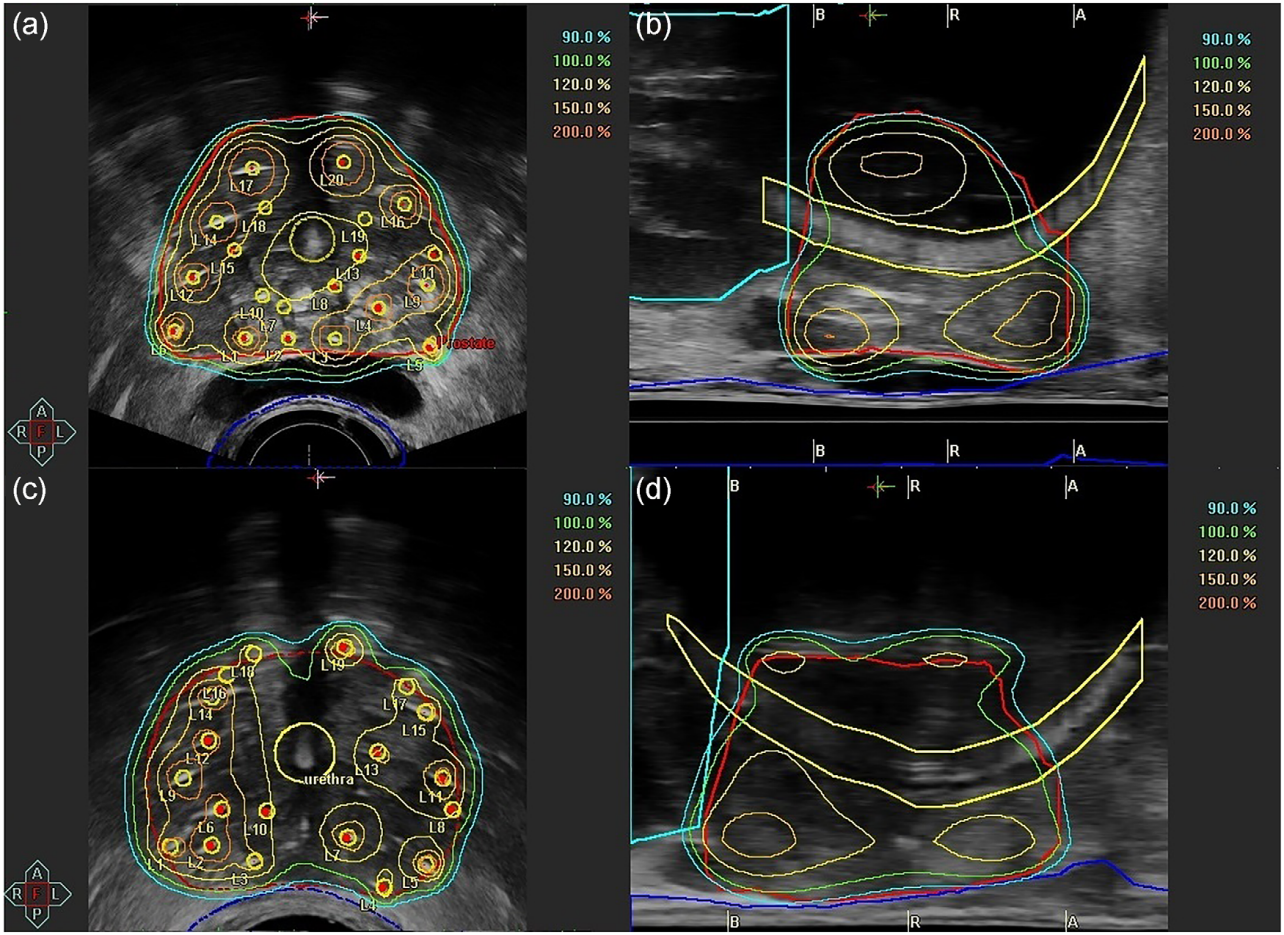
(a) Axial TRUS image showing a representative dose distribution in a patient who received an HA rectal spacer, with the rectum (blue contour) displaced posteriorly away from the high‐dose region. (b) Sagittal TRUS image of the same case, showing the 90%–200% isodose lines for a prostate HDR treatment with a 15 Gy prescription dose. (c) Axial TRUS image showing a representative dose distribution in a patient with a prostate‐to‐anterior rectal wall distance of less than 5 mm who did not receive HA rectal spacer injection; the anterior rectal wall received a higher dose due to its close proximity to the prostate. (d) Corresponding sagittal TRUS image through the urethra (yellow contour) and rectum (blue contour).

More recent investigations have further highlighted the relationship between prostate–rectal separation and rectal dosimetry during HDR brachytherapy. Beaudry et al. recently reported that rectal spacers increased average prostate–rectal separation from approximately 4 mm to nearly 8 mm and significantly reduced rectal dose while maintaining comparable prostate coverage during HDR boost brachytherapy. Their simulation analysis suggested that prostate–rectal separations greater than 4 mm were associated with substantial rectal dose reduction and improved ability to achieve dosimetric constraints.^28^ Our findings are consistent with these observations and further suggest that same‐day HA spacer placement may be particularly useful in patients with baseline prostate–rectal separation less than 5 mm, where achieving adequate rectal sparing without compromising target coverage can be technically challenging. Figure 5 shows a representative dose distribution comparison between cases with and without spacer placement. In the case with a prostate‐to‐anterior rectal wall distance of less than 5 mm, the patient did not receive HA rectal spacer injection, and the anterior rectal wall received a higher dose due to its close proximity to the prostate.

**FIGURE 6:**
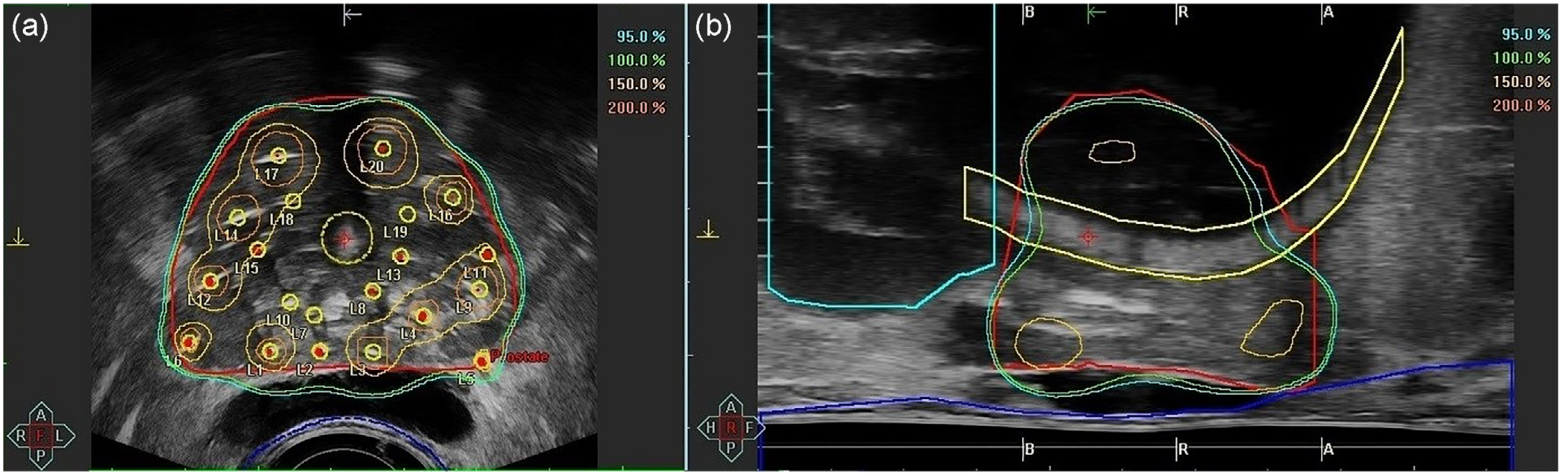
(a) An axial section of a TRUS image, showing the HA rectal spacer displacing the rectum (blue line) posteriorly, away from the high dose region. (b) A sagittal section of the same image. For a 15 Gy prescription dose, 95%–200% isodose lines are shown for prostate HDR treatment.

Although differences in prostate V150 and V200 were not statistically significant in our study, the observed trend toward reduced high‐dose volumes suggests that spacer placement may contribute to improved dose homogeneity within the target. The increased separation between the anterior rectal wall and prostate may also influence catheter geometry and dose distribution. Greater prostate–rectum distance may allow catheters to be positioned further from the prostatic urethra without increasing radiation dose to the rectum. This, in turn, may contribute to reduced urethral dose while maintaining adequate target coverage, particularly in patients with smaller anterior–posterior prostate dimensions. A representative isodose distribution illustrating this effect is shown in Figure 6.

The placement of a rectal spacer elevated the prostate anteriorly, potentially leading to pubic arch obstruction. Depending on the shape and size of the prostate, the amount of rectal spacer injected needed to be adjusted to maximize rectal dose reduction while ensuring that prostate elevation did not obstruct catheter placement and HDR brachytherapy dose delivery. In contrast to PEG hydrogel spacers, which require mixing of precursor solutions prior to injection,^29^ the HA spacer is delivered as a preformed gel.^22^ This allows flexibility in the volume administered based on intra‐procedural assessment. In our cohort, two patients received 6 cc of HA prior to catheter placement, while the remaining patients received 3 cc. Additional HA was administered following HDR brachytherapy to achieve a total injected volume of 9 cc in all patients, with the goal of maintaining rectal separation during subsequent EBRT.

An important strength of this study is the demonstration that same‐day HA spacer placement preserved TRUS image quality and enabled a fully real‐time TRUS‐guided HDR brachytherapy workflow without requiring CT‐based planning or a separate spacer placement procedure. Unlike PEG spacers, which may introduce air‐related artifacts and degrade ultrasound visualization immediately after injection, the HA spacer maintained adequate acoustic transparency for prostate visualization, catheter placement, contouring, and treatment planning. This approach eliminates the need for additional procedures or patient transfers between implantation and treatment planning, potentially improving workflow efficiency while minimizing the risk of catheter displacement.

This study has a few limitations. First, the analysis includes a relatively small number of patients from a single institution, which may limit generalizability. Second, the study was not randomized, and patients were selected for spacer placement based on prostate–rectal wall distance, introducing the potential for selection bias. Third, the comparison group consisted of patients treated without spacer placement rather than a prospective matched cohort. Finally, this analysis focused primarily on dosimetric outcomes, and longer follow‐up will be required to determine whether the observed reductions in rectal dose translate into clinically meaningful reductions in gastrointestinal toxicity. Future studies with larger patient cohorts and prospective study designs are warranted to further validate these findings.

## 5 CONCLUSIONS

Our clinical workflow demonstrates that HA rectal spacer injection immediately prior to TRUS‐guided HDR PBT is feasible and preserves ultrasound image quality, enabling real‐time treatment planning without the requirement for CT‐based imaging. Same‐day HA spacer placement increased the separation between the prostate and rectum and was associated with reduced rectal V75 while maintaining excellent prostate target coverage in patients with limited baseline prostate–rectal wall separation. Although the absolute dosimetric differences were modest, these findings are clinically relevant in anatomically challenging patients in whom prostate dose coverage might otherwise need to be compromised to satisfy rectal dose constraints. In addition, the ability to perform spacer placement, catheter implantation, treatment planning, and HDR delivery within the same procedure represents a practical advantage of the HA spacer workflow.

## AUTHOR CONTRIBUTIONS

Sook Kien Ng and Omar Ishaq conceptualized and designed the study. Sook Kien Ng collected and analyzed the data. Sook Kien Ng drafted the manuscript. Damodar Pokhrel, Colin Huang, Francis Asamoah, Jordon Holmes, Arpon Prabhu, and Omar Ishaq contributed to study design and provided clinical oversight. Colin Huang, Francis Asamoah and Omar Ishaq assisted with data interpretation and technical implementation. All authors reviewed, revised, and approved the final manuscript.

## CONFLICT OF INTEREST STATEMENT

The authors declare no conflicts of interest.

## ETHICS OR COMPLIANCE STATEMENT

This study was approved by the Institutional Review Board of Indiana University (IRB protocol number: 24408).

## Data Availability

The data that support the findings of this study are available from the corresponding author upon reasonable request.
